# Patient Views Around Their Hernia Surgery: A Worldwide Online Survey Promoted Through Social Media

**DOI:** 10.3389/fsurg.2021.769938

**Published:** 2021-12-24

**Authors:** Barbora East, Susannah Hill, Nicola Dames, Sue Blackwell, Lynn Laidlaw, Hakan Gök, Cesare Stabilini, Andrew de Beaux

**Affiliations:** ^1^Department of General Surgery, Royal Infirmary of Edinburgh, Edinburgh, United Kingdom; ^2^3^rd^ Department of Surgery, Motol University Hospital, Prague, Czechia; ^3^Patient Representative, London, United Kingdom; ^4^Patient Representative, Glasgow, United Kingdom; ^5^Patient Representative, Liverpool, United Kingdom; ^6^Hernia Istanbul®, Hernia Surgery Center, Istanbul, Turkey; ^7^DISC (Department of Surgical Sciences), University of Genoa, Genoa, Italy

**Keywords:** hernia, patient information, patient involvement, PROMs, surgical mesh, surgical outcomes, hernia registry

## Abstract

**Introduction:** Hernias are one of the most common surgical diagnoses, and general surgical operations are performed. The involvement of patients in the decision making can be limited. The aim of this study was to explore the perspectives of patients around their hernia and its management, to aid future planning of hernia services to maximise patient experience, and good outcomes for the patient.

**Methods:** A SurveyMonkey questionnaire was developed by patient advocates with some advice from surgeons. It was promoted on Twitter and Facebook, such as all found “hernia help” groups on these platforms over a 6-week period during the summer of 2020. Demographics, the reasons for seeking a hernia repair, decision making around the choice of surgeon, hospital, mesh type, pre-habilitation, complications, and participation in a hernia registry were collected.

**Results:** In total, 397 questionnaires were completed in the study period. The majority of cases were from English speaking countries. There was a strong request for hernia specialists to perform the surgery, to have detailed knowledge about all aspects of hernia disease and its management, such as no operation and non-mesh options. Chronic pain was the most feared complication. The desire for knowledge about the effect of the hernia and surgery on the sexual function in all age groups was a notable finding. Pre-habilitation and a hernia registry participation were well-supported.

**Conclusions:** Hernia repair is a quality of life surgery. Whether awaiting surgery or having had surgery with a good or bad outcome, patients want information about their condition and treatment, such as the effect on aspects of life, such as sex, and they wish greater involvement in their management decisions. Patients want their surgery by surgeons who can also manage complications of such surgery or recommend further treatment. A large group of “hernia surgery injured” patients feel abandoned by their general surgeon when complications ensue.

## Introduction

Hernias are a common affliction worldwide. The main treatment option is surgical repair of the hernia. Much of the research related to hernia surgery focusses on the short- and longer-term outcomes, such as recurrence and more recently chronic pain. Patient reported outcome measures (PROMs) have gained popularity, but PROMs still only ask questions to patients that the doctor wants to be answered (1). Such questions may not reflect the priorities of the patient, or indeed address elements of the patient experience or barriers to their treatment or what best leads to satisfying outcomes. Such failures in seeking patient focussed data are not unique to hernia surgery (2). However, patient focussed priorities are a key part of the GRADE approach in writing the clinical guidelines (3). It dictates: “*to make sensible recommendations guideline panels must consider all outcomes that are important or critical to patients for decision making”*. However, there is a lack of research or knowledge about what is important or critical to our patients? Two recent studies exploring some of these issues in ventral hernia repair recruited only 22 and 30 patients, respectively, in single centres, making their generalisability less clear cut (4, 5).

An additional area of concern in hernia surgery is the steady growth in mesh related litigation and the increasing number of patient support groups focused on problems possibly related to their mesh. There is a clear need to open discussion with such groups to understand better and thus heal this surgeon—patient relationship. Despite that, some surgeons are reluctant to accept responsibility over implants they use or have insufficient knowledge about their properties or do not inform their patients well about all the risks and benefits of mesh or indeed non-mesh options. Not unsurprisingly, a growing body of patients are losing trust in the surgical industry. Online surveys are good tools to utilise in such situations when large numbers of patients need to be involved (6).

The aim of this study was to explore and gain insights into the perspectives of patients related to their hernia and its management through an online based survey, promoted through social media.

## Methods

The research group consisted of eight people. An initial meeting was held online and consisted of the principal researcher, another three surgeons with a major hernia interest, two patient representatives regularly attending hernia meetings, and two more patient representatives, one of which has re-trained as a nurse and the other with expertise in facilitating the patient groups.

At the initial meeting, the investigators agreed on the main aims of the survey—to investigate expectations of patients from hernia surgery focussing on their priorities throughout the process of hernia surgery—initial motivation for repair, preoperative consultation, operative and follow-up preferences, along with their views about mesh use. The study questions were composed and over a period of 4 weeks, the questions were refined in terms of readability in the English language, using the SurveyMonkey platform.

The 42-questions questionnaire was launched on social media. Posts using Twitter on the personal profiles of the investigators and on @EuroHerniaS and @ColostomyUK with a link to the survey were retweeted regularly. Posts on Facebook using similar profiles were undertaken. In addition, all the patient support groups that contained the word “hernia” or “mesh” were approached with a request to share the link to the survey with their members. The groups are listed in Table 1 comprising a total of 29,697 members. A number of groups refused to either accept the principal investigator and/or share the link to the survey and these are listed in the Table 2. The survey was promoted over a 6-week period during June and July 2020. Link to the survey was promoted by all the authors through their Twitter social media feed, with regular retweets. The survey questions can be viewed at http://www.surveymonkey.com/r/WhatDoPatientsWantFromHerniaOperation.

**Table 1:** Facebook groups approached who were willing to disseminate the survey with membership number in each group.

**Name of the group**	**Number of members**
Hernia Mesh Australia Support Group	873
Mesh Unity	166
Inguinal Hernia Surgery Sucks	182
Say No to hernia Mesh	38
Hernia and Obesity Pats Group	129
No Mesh Hernia Surgery	31
Mesh UK Charitable Trust: Support Group	1,800
Hérnia Inguinal, Incisional, Umbilical e Casos Complexos—SP	1,100
Hernia awareness Movement / Mesh Victims United	80
Mesh problems	3,400
Hernia/pelvic mesh Support Group Australia	72
Hernia Inguinal	601
Mesh Removal Patients (Hernia And Pelvic)	1,200
Incisional Hernia Support group	771
MESH.	2,700
Hernia Mesh Hurts Too (A Support Group for All Mesh Injured)	862
Surgical Mesh Complication Information: Hernia Mesh, Transvaginal Mesh, …	933
Mesh problems	3,400
Say No To Hernia Mesh	38
Mesh Angels	972
LIVE LIFE MESH FREE @JanUrban12	699
Mesh Awareness Movement—(MAM)/Mesh Victims United	1,100
umbilical hernia support	1,500
Sports Hernia/Core Muscle Injury Awareness and Support	770
Meshed up by mesh (England)	283
Mesh.CANADA	212
Susan's Incisional Hernia Support Group	25
Mesh Australia support group 2	64
Links on Mesh and other Problem Medical Devices	1,800
Mesh Angelz Support Group	170
Hernia Mesh Complications Support Group	93
Ban Plastic Mesh!	25
Hernia Mesh Complication support group	2,700
Mesh Sling South Africa	46
Scottish Mesh Survivors	700
Mesh victims south Africa	162
**Total number of members**	**29,697**

**Table 2:** Facebook groups approached who were NOT willing to disseminate the survey with membership number in each group.

**Name of the group**	**Number of members**
Mesh removal—Stories and surgery info	1,200
Sling the mesh	8,700
Hernia Mesh AU	72
Sling the mesh N Ireland	582
Mesh Down Under Support Group	973
Hernia Mesh awareness NI	254
Mesh Awareness Wales	42
Action for Mesh Injured Patines	165
WELSH MESH SURVIVORS	236
Mesh Awareness Movement—(MAM)/Mesh Victims United	1,100
Kugel Mesh Hernia SURVIVORS FORUM	194
Pelvic and Hernia Mesh Removal in Australia	2
Hernia Mesh Pros And Cons Discussion Group	263
My hernia mesh story international.	22
HERNIA/PELVIC MESH SUPPORT GROUP WA, AUST.	72
Mesh Medical Device News Desk	1,500
HERNIA SURGERY	93
My Natural Hernia Cure Group	545
Inguinal Hernia Survivors	381
Hernia group	21
Canadian Victims Of Hernia And Transvaginal Mesh	104
Advanced Hernia Solutions (AHS)	25
Mesh Ireland	119
**Total number of members**	**16,665**

It was anticipated that the findings of the survey would be presented as descriptive data, and no statistical analysis was planned.

## Results

In total, 397 people who have or have had hernia surgery completed the survey. The age, sex, country/region of residence, type of hernia, and the number of previous repairs where applicable are given in Table 3. On reviewing the responses, three groups of patients were identified, those who had a hernia repair and were happy with the outcome *n* = 112 (Group A), those who have had surgery but were unhappy with their outcome *n* = 176 (Group B), and those with a hernia awaiting surgery *n* = 105 (Group C). Four patients did not express whether they were happy or unhappy with the procedure they have undergone. The age, sex, average number of hernia repairs, presence of recurrence, and ongoing complication between Groups A and B are given in Table 4. Of note, patients who are unhappy following hernia surgery are more likely to have a recurrence of their hernia and/or have an ongoing complication of their hernia surgery and were more likely to be women.

**Table 3:** Basic demographics of the respondents.

**Sex**		**Age**		**Country/Region**		**Type of hernia**		**No. of previous repairs**		**Satisfaction**	
Male	151	18–25	4	Australia	45	Incisional	109	0	105	Happy	112
Female	243	26–35	24	Continental Europe	22	Inguinal	145	1	178	Unhappy	176
Didn't say	3	36–45	77	South America, Africa, Asia	12	Epigastric	10	2	54	Not answered	4
		46–55	127	UK, Ireland	177	Femoral	7	3 and more	60	N/A	105
		56–64	109	Canada, USA	133	Diaphragmatic	3				
		65–74	47	Unknown	8	Parastomal	62				
		75+	9			Umbilical	74				
						Other	9				
						Ventral	1				
						Unknown	7				
						Total:	427				

*Satisfaction with treatment was only applicable to those already having had a hernia repair*.

**Table 4:** Basic demographics of Groups A, B, and C.

**Group**	**A**	**B**	**C**	
**Satisfaction**	**Happy**	**Unhappy**	**N/A**	**No answer**
No.	112	176	105	4
Incisional (78)	33 (41.3%)	45 (56.3%)	29	1
Inguinal (80)	42 (34.7%)	78 (64.5%)	21	1
Parastomal (34)	19 (55.9%)	15 (44.1%)	28	
Ventral (59)	18 (30.5%)	39 (66.1%)	25	2
Average no. of repairs	1.4	1.7		
Hernia recurrence	20.5%	35.8%		
Ongoing complication	35.8%	73.3%		
Female	59 (52.7%)	114 (64.8%)	68 (64.8%)	
Male	53 (47.3%)	60 (34.1%)	36 (34.3%)	
Unknown	0	2	1	
Average age	52	52.3	52.5	
North America	41 (36.6%)	60 (34.1%)	30 (28.6%)	
UK	49 (43.8%)	65 (36.9%)	56 (53.3%)	
Europe	9 (8.0%)	14 (8.0%)	5 (4.8%)	
AUS+NZ	8 (7.1%)	28 (15.9%)	8 (7.6%)	
Rest of the world	2 (1.8%)	4 (2.3%)	6 (5.7%)	

*Group A: patients satisfied with their hernia repair. Group B: patients with decisional regret. Group C: patients that did not have surgery at the time of completing the survey*.

The reasons why patients seek medical help with their hernias are shown in Table 5. Pain, a bulge, and limitation during sports and other activities are the commonest reasons.

**Table 5:** Reason to have a hernia operation by hernia type and by the patient group.

	**Bulge**	**Inability to do sports**	**Limitation at work**	**Pain**	**Dietary restriction**	**Sexual dysfunction**	**Cosmesis**	**No symptoms**	**Stoma bag not fitting**	**Problems with digestion**	**Other**
Overall	41	39	21	56	12	15	31	4	N/A	27	5
Incisional	45	42	25	54	17	13	46	3	N/A	39	7
Inguinal	34	49	25	55	10	23	8	3	N/A	18	6
Parastomal	47	24	11	65	18	5	48	2	**50**	32	0
Ventral	49	35	17	55	10	13	39	2	N/A	27	4
Group A	**54**	46	20	50	8	8	29	2	N/A	21	3
Group B	**28**	39	24	66	16	22	24	5	N/A	34	6
Group C	49	31	18	43	10	10	44	4	N/A	24	4

*Values are given as percentage values of those who responded to this question and were in each respective group. Respondents were given the advice to select the three most important reasons for repair, however, many have ticked more than 3 and many selected all offered options. Among “other” were in the free text mentioned things like fear, mental health issues, and then reasons for mesh removal like autoimmune disease or rejection. Group A: happy patients post hernia repair. Group B: unhappy patients post hernia repair. Group C: patients that have not had an operation to date*.

The ability to choose the medical facility was not applicable to 27% (105/393) of the respondents. Out of the remaining 289 patients, 27% would go to their local hospital, 27% to a hernia centre, 25% to a private facility, and 14% to a large university hospital. The remaining patients would either follow their preferred surgeon, recommendation of other patients, or find a facility that provides solely non-mesh repairs.

The ability to choose their surgeon was not applicable to 30% (121/397) of the respondents, and 22% (88/397) did not share their preferences (Table 6). Only 1.3% (5/308) of respondents saw it as an advantage for their surgeon to be involved in hernia research. About 60% of patients would like to be operated by a hernia specialist irrespective of the number of repairs they have undergone, type of repair they would prefer, total complication rate, or satisfaction with previous hernia operation. However, in the group that would prefer a hernia specialist, there were more patients with an ongoing complication than in the other group. In the group of patients that would prefer a non-mesh repair, there were less patients that were happy with previous repairs in comparison with the group that would be willing to receive a mesh.

**Table 6:** The choice of patients about their surgeon.

	**Wish for a specialist**	**No need for specialist**	**Non-mesh technique**	**OK with mesh**
No	186 (60.4%)	122 (39.6%)	122 (39.6%)	186 (60.4%)
Average no. of repairs	1.3	1.3	1.2	1.2
Recurrence	39 (29.3%)	28 (30.1%)	26 (28.0%)	41 (44.1%)
Non-mesh repair	76 (40.9%)	46 (37.1%)	N/A	N/A
Complication (total)	82 (61.7%)	54 (58.1%)	58 (67.4%)	78 (55.7%)
Resolved complication	14 (10.5%)	12 (12.9%)	7 (8.1%)	19 (13.6%)
Ongoing complication	68 (51.1%)	42 (45.2%)	51 (59.3%)	59 (42.1%)
Already had a repair	133 (129 answered)	93	86 (84 answered)	140 (138 answered)
Satisfied	54 (41.9%)	38 (40.9%)	22 (26.2%)	70 (50.7%)

*Percentage values of recurrences and complications rates were calculated out of those who had an operation only. About 60% of patients would like to be operated by a hernia specialist irrespective of the number of repairs they have undergone, type of repair they would prefer, total complication rate, or satisfaction with previous hernia operation. However, in the group that would prefer a hernia specialist, there were more patients with an ongoing complication than in the other group. In the group of patients that would prefer a non-mesh repair, there were less patients that were happy with previous repairs in comparison to the group that would be willing to receive a mesh*.

Prior to any hernia surgery, there is a strong desire for a detailed explanation around how any operation was going to impact their quality of life (98%), to be told everything about potential complications (99%), such as any impact on their sexual function (84%), to be able to discuss all options with the surgeon and become part of a joint decision process (97%), and to be told what happens if they chose no operation at all (98%). Approximately 97% wanted to be operated by the same person as whom they have seen at the initial appointment. About 85% would appreciate a patient information booklet to be able to educate themselves on their diagnosis, 67% were keen to be involved in a patient support group and being spoken to in a language they understand was important to 98%. However, when asked in a separate question, 9% of respondents agree with the statement that they do not need to be told anything and will fully trust their surgeon which is conflicting with their previous replies.

The choice of operation was also important to the respondents. About 71% of patients would rather have a more complex or difficult operation that minimised the risk of recurrence while 2.5% were happy for a “quick easy operation” that might only last a few years. The rest reported that the choice of operation depends on many factors, and they needed to know more or mention their request for a mesh not to be used.

The preferred type of operation by the three study groups is given in Table 7. In addition to surgical options, the mesh used was also questioned. About 92% of respondents expect their surgeon to explain to them the pros and cons of various types of the mesh before their operation. When asked about their “ideal” mesh, 41% answered that they would prefer a non-mesh option, 26% would like to select one based on an interview with the surgeon, and only 20% are happy for their surgeon to select one for them. The remaining 14% were split between those who would accept a standard mesh, would like a “new, modern” mesh, biodegradable, biological, resistant to infection, or visible on a CT scan mesh. Only 5/394 respondents mentioned the word “safe.” In the group of parastomal patients who have already had a repair (*n* = 34), only 6% would prefer a non-mesh option. And overall, only 8% of respondents are either not interested in knowing about or do not want to be told about non-mesh options.

**Table 7:** Choice of operative approach by group (MIS—minimally invasive approach).

**Group**	**A**	**B**	**C**
**Satisfaction**	**Happy**	**Unhappy**	**Not operated yet**
Surgeons preference	64 (57.1%)	31 (18.0%)	38 (37.3%)
Open repair	21 (18.8%)	34 (19.8%)	15 (14.7%)
MIS	22 (19.6%)	46 (26.7%)	30 (29.4%)
Safest	1 (0.9%)	7 (4.1%)	1 (1.0%)
Non-mesh repair	1 (0.9%)	20 (11.6%)	3 (2.9%)
Joint decision	3 (2.7%)	5 (2.9%)	5 (4.9%)
Other	0	1 (0.6%)	0
No operation	3 (2.7%)	28 (16.3%)	11 (10.8%)

The majority of respondents would like to be offered a pre-habilitation programme (78%) and 76% would be willing to change their lifestyle prior to the operation had they been told it could improve their outcomes (Table 8). In total, 395 people answered both questions about satisfaction with their hernia repair and the possibility to change their lifestyle. It was found that 292 of those had already undergone a hernia repair. About 50% of those who had a repair and were not going to change their lifestyle are happy with their previous repair. In the group willing to change their lifestyle to improve outcomes, only 37% are happy with the results of the previous surgery (Table 9).

**Table 8:** The willingness of patients to change their lifestyle prior to their operation, and take part in pre-habilitation.

**Group**	**Satisfaction**	**No**.	**Willing to change lifestyle**	**%**	**Not willing to change lifestyle**	**%**	**Unsure**	**%**
A	Happy	112	79	70.5	6	5.4	27	24.1
B	Unhappy	176	137	77.8	5	2.8	34	19.3
C	N/A	105	82	78.1	16	15.2	7	6.7
	Unknown	4						

**Table 9:** The willingness of patients to change their lifestyle and take part in pre-habilitation vs. reported happiness with their surgical outcome in those who had already undergone one or more operations to repair their hernia.

**Group**	**No**.	**%**	**Operated**	**Happy**	**%**
NOT willing	19	4.8	12	6	50
Don't know	77	19.5	62	27	43.5
YES willing	299	75.7	218	81	37.2

The complications of hernia surgery that were most feared by the respondents are given in Table 10 by the study group.

**Table 10:** Complications most feared after hernia surgery.

	**Group**	**A**	**B**	**C**	
**Feared complication**	**Total** **397**	**Happy** **112**	**Unhappy** **176**	**Not operated** **105**	**Not answered** **4**
Recurrence	269 (67.8%)	89 (79.5%)	103 (58.5%)	76 (72.4%)	
Chronic pain	277 (69.8%)	67 (59.8%)	141 (80.1%)	67 (63.8%)	
Sexual dysfunction	87 (21.9%)	19 (17.0%)	49 (27.8%)	20 (19.0%)	
Inability to work	98 (24.7%)	19 (17.0%)	60 (34.1%)	21 (20.0%)	
Inability to exercise	115 (29.0%)	29 (25.9%)	58 (33.0%)	28 (26.7%)	
Dietary restriction	34 (8.6%)	4 (3.6%)	24 (13.6%)	6 (5.7%)	
Cosmesis	25 (6.3%)	3 (2.7%)	11 (6.3%)	11 (10.5%)	
Major complications	256 (64.5%)	68 (60.7%)	121 (68.8%)	67 (63.8%)	
Other	20 (5.0%)	5 (4.5%)	13 (7.4%)	2 (1.9%)	

*Respondents were advised to mark up to three most feared complications, however, many have marked all options and added a few more like mesh rejection and possible mesh related autoimmune disease*.

Seeing their surgeon again on the morning of surgery was important to 90% of respondents. In terms of anaesthesia, 7% of respondents would prefer to be awake during the operation, the others either prefer general anaesthesia or will follow the recommendation of the surgeon and/or anaesthetist. Following surgery, 97% of respondents wanted to speak to the surgeon and be able to discuss with them the details of their repair. The majority of respondents (302/390) (77%) are prepared to stay in hospital or be at home as long as it takes to maximise the success of surgery. However, for some patients, return to normal activities (13%), sports (6%), or work (6%) is a priority. Information on how to minimise their risk of hernia recurrence was important to 96%, while 27% would be willing to accept a small recurrence if it did not hurt or enlarge.

Patients with no decisional regret about the operation have reported higher satisfaction with the behaviour of surgeon to the group B. About 93% of respondents would like to be part of a hernia registry and 62% of patients would feel better if they had a planned follow-up for at least 2 years.

## Discussion

This worldwide, online survey of people with a hernia, some of whom have already had at least one hernia repair, has identified several important reminders for surgeons involved in the management of hernias. Patients are looking for their surgeon to be an expert in hernia surgery, and able to spend time to explain their options—which is a discussion of the benefits, risks, and alternatives, such as operation vs. no operation, open vs. laparoscopic, mesh vs. non-mesh repair, and when mesh used, the various mesh options. Linked to all of this was the biggest fear after surgery of chronic pain. Communication with their surgeon during their time in hospital was seen as important by nearly every respondent irrespective of age, sex, nationality, type of hernia, or type of preferred repair. While the idea of a registry was supported by nearly all the respondents, a smaller proportion were willing to return for routine follow up.

The survey was heavily promoted on social media by the investigators with support from the European Hernia Society social media channels. We acknowledge that the majority of respondents are from English speaking countries and were obviously active on Twitter, leading to some bias in our study group. Furthermore, the principal investigator (BE) contacted as many hernias help and mesh help groups that we could identify (Tables 1, 2). While there was some concern that responses from these groups might skew the data, it was considered that those with a problem may be more motivated to reply to the survey to help improve the situation for others. Nevertheless, there was a strong consensus in the responses to the questions between those who had a hernia repair vs. those who had not, and those who already had surgery with a good outcome compared with a poor outcome. Several hernias help groups refused to have any communication with us. Others were more receptive but had elements within them that replied on social media with quite disturbing messages. There was a high level of mistrust expressed and the principal investigator was often subjected to offensive comments especially when surgical mesh was discussed. In most groups, there were well-educated and articulate patient representatives that have been very helpful through the process of distributing the survey, allaying fears, and encouraging followers to take part. Despite these groups having tens of thousands of members, only 397 responded to the survey in the study period, even when communication had taken place about the reasons for this patient focussed study that leaves us to a question how many of those are actually active. Nevertheless, one of the respondents has summarised it well and has agreed to have their quote and name published. “I would say they want to be fixed and not suffer a far more serious fate because of what was used to repair their hernia. Get some more bloody specialists and get hernia repair out of the hands of a general surgeon.” (Gary McCollom – Canada). While the evidence for hernia specialisation for primary inguinal and ventral hernia is not strong (7), nor is evidence for a hernia volume related improved outcomes (8), the feedback was more about the handling of the situation when something went wrong, in particular chronic pain. The general surgeon seemed to have nothing to offer and discharged the patient who still had their problem and did not know where to go for help. Indeed, this was one of the main take home messages for the investigators which came from the free text answers left by 123 respondents. The second main message was the lack of knowledge about hernias in the medical profession. Several respondents mentioned that they had been told to not work, or lift, or exercise with a hernia by their doctor, often in contradiction to recommended practise (9). Additionally, one patient claimed to have been told while she was pregnant that her umbilical hernia required urgent repair despite no symptoms.

It is clear that patients want knowledge about their condition, their options, and the likely outcomes. It is this knowledge that leads to informed consent. While there are legal implications around the need for informed consent, the primary aim of such interaction is to help the patient choose the right option for them and inform them of all materials used (10). Other research groups have focused on the communication between medical professionals and patients pointing out the need to improve the process of informed consent prior to hernia surgery. One audit of informed consent documents in patients undergoing inguinal hernia repair noted that only 66% of them contained all the common or serious complications (11). There are challenges to informed consent, although we understand more about the process including the requirements when producing written information (12). The present study strongly reiterates the need for detailed patient-surgeon discussion (13). Keeping in mind that the recollection and understanding of patient of the information given during the consent process can be variable (14), it is clear that there needs to be more effort spent on giving information and documenting what was given (15). In our survey, we had many comments from the respondents about not being told that mesh was involved in the repair, or not having other surgical options mentioned, but we have no way how to validate these statements. However, it was mentioned by many patients that general surgeons are no longer trained in non-mesh repairs, in particular for inguinal hernias. There are serious issues for training the hernia surgeons for the future (8), and the need to be able to offer non-mesh options in certain patient and hernia types (16).

Pre-habilitation, especially for more complex abdominal wall repair is another area of study. In this survey, two-thirds of respondents were willing to make some lifestyle changes prior to their operation. We acknowledge that this proportion was only a theoretical agreement in an anonymous survey, and the real-world commitment to pre-habilitation may be not so high. Varying literacy amongst patients with hernias may lead to many having unrealistic expectations (7). It is estimated that in the United States, about 80% of patients have modifiable risk factors prior to ventral hernia repair. Due to low health literacy, 20% have wrong self-assessment and believe they are in a better condition than they really are and only one-third see no barriers in joining pre-habilitation programme (17). Another study used a small focus group of 22 patients to explore their motivation for surgery and expectations after recovery (4).

There was a very strong consensus that patients wished to be operated on by the same surgeon they had met during their appointment and it was also important to them that this surgeon knew what they were doing—a hernia specialist. Only 4% did not mind being operated by someone else. This is an important point for healthcare organisers and should be taken into consideration when organising the surgical practise.

When asking patients actively to report adverse outcomes, the reported incidence of such events can increase significantly (18). A study looked at patient satisfaction after groin hernia repair in 373 adult patients with a special focus on pain and follow-up (19). For patients who were well, there was a reluctance to attend follow-up. We have observed similar views in our survey. While the idea of a registry was supported by nearly all the respondents, a smaller portion was willing to return for routine follow-up. However, other methods can be used to gain follow-up patient information, smart phone Apps, and online reporting tools. The involvement of patients in what to measure is also important. The type of outcomes perceived as negative by surgeons' changes from strictly defined items like recurrence to much broader ones like quality of life. Hubbard et al. have addressed the lack of patient-led research priorities, and this was confirmed by Alawadi et al. demonstrating the importance of understanding the perspective of patients and addressing their specific needs prior surgery also (7, 20). The outcomes that surgeons have measured in the past, are not as important to respondents while other outcomes can have greater weight to them. In this survey, sexual function post hernia repair was mentioned by the majority of our respondents in all the age groups (81% in the group above 65 years of age).

Many patients see “mesh” as the cause of all their problems. For some, this may be the case, but many of the issues described to us, surgical error, lack of pre-optimisation for the surgery, poor communication about complications, and lack of follow-up or care for a complication caused by the operation was more the problem. Mesh-injured is a misnomer, and perhaps should be replaced with “hernia surgery injured.” Many of the people in these support groups are upset with the whole medical industry, often left with debilitating pain and other complications with no one to reach for help, repeatedly rejected by multiple specialists, and often in significant financial difficulties due to the medical state they got in. One gentleman in South Africa who had a laparoscopic IPOM for a small umbilical hernia resulting in multiple bowel obstructions and numerous attempts at mesh removal claimed to have racked up a 400,000 USD debt to his surgeon and has not been able to work ever since. We do not know why all the remedial operations had to be done robotically and the technical standard of the procedure, but “the mesh” was blamed, perhaps not correctly.

It is easy to assume that the increasing number of recurrences and complications leads to an unhappy patient. While there is some truth to this, the stoma patient group in this study actually demonstrated the opposite. This group as a whole had the highest number of complications and yet contained the highest number of patients that were satisfied. Further research to unravel the reasons for this is needed. About 6% of those with a parastomal hernia said they would “prefer no mesh” vs. 52% for inguinal. Communication and trust in their surgeon may be a factor in this study. Detailed analysis by hernia type was not undertaken as such data dredging with increasing smaller numbers (as noted in Table 4) was considered scientifically unsound.

Having undertaken this work, we are left with a clear view that while the collection of surgical outcome data by every hernia surgeon is important, the collection of quality of life outcome data, reported by patients is even more important. There is a need to engage further with our patients, and refine quality of life measurements that measure what our patients want us to measure as important to them. The face-to-face patient focus groups, social media campaigns, with appropriate sociology validation of the quality of life tools will all be important in this regard. In addition, hernia registries that include patients undergoing watch and wait, with life-long follow-ups, such as data from patients, other healthcare professionals, utilising smart phone Apps, and emerging machine reading and artificial intelligence of healthcare medical records technology, will add to the knowledge of what management pathway is the best option for each individual patient with a hernia.

## Conclusion

Within the bias of our patient population recruited *via* Twitter, we have found a desire for patients to be looked after by hernia specialists where possible. Elements around quality of life after surgery is their main outcome measure. And if something goes wrong after surgery, they demand to be listened to and have services in place to manage them. Mesh-injured should be replaced by hernia-surgery-injured in the future for clearer communication. Greater patient involvement is needed to help develop hernia services.

## Data Availability Statement

The raw data supporting the conclusions of this article will be made available by the authors, without undue reservation.

## Ethics Statement

Ethical review and approval was not required for the study on human participants in accordance with the local legislation and institutional requirements. Written informed consent for participation was not required for this study in accordance with the national legislation and the institutional requirements.

## Author Contributions

BE: study concept, literature review, survey questionnaire preparation, analysis of data, manuscript writing, revision of the manuscript, and final approval of the manuscript. SH, ND, SB, and LL: survey question preparation and analysis of data. HG and CS: survey questionnaire preparation, analysis of data, and manuscript writing. AB: literature review, survey questionnaire preparation, analysis of data, manuscript writing, revision of the manuscript, and final approval of the manuscript. All authors contributed to the article and approved the submitted version.

## Conflict of Interest

The authors declare that the research was conducted in the absence of any commercial or financial relationships that could be construed as a potential conflict of interest.

## Publisher's Note

All claims expressed in this article are solely those of the authors and do not necessarily represent those of their affiliated organizations, or those of the publisher, the editors and the reviewers. Any product that may be evaluated in this article, or claim that may be made by its manufacturer, is not guaranteed or endorsed by the publisher.
